# Long-Term Efficacy and Safety of Pemafibrate, a Novel Selective Peroxisome Proliferator-Activated Receptor-α Modulator (SPPARMα), in Dyslipidemic Patients with Renal Impairment

**DOI:** 10.3390/ijms20030706

**Published:** 2019-02-06

**Authors:** Koutaro Yokote, Shizuya Yamashita, Hidenori Arai, Eiichi Araki, Hideki Suganami, Shun Ishibashi, on Behalf of the K-877 Study Group

**Affiliations:** 1 Department of Diabetes, Metabolism and Endocrinology, Chiba University Hospital, Chiba 260-8670, Japan; 2 Department of Endocrinology, Hematology and Gerontology, Chiba University Graduate School of Medicine, Chiba 260-8670, Japan; 3 Department of Community Medicine and Department of Cardiovascular Medicine, Osaka University Graduate School of Medicine, Osaka 565-0871, Japan; shizu@imed2.med.osaka-u.ac.jp; 4 Rinku General Medical Center, Osaka 598-8577, Japan; 5 National Center for Geriatrics and Gerontology, Aichi 474-8511, Japan; harai@ncgg.go.jp; 6 Department of Metabolic Medicine, Faculty of Life Sciences, Kumamoto University, Kumamoto 860-8556, Japan; earaki@gpo.kumamoto-u.ac.jp; 7 Clinical Data Science Department, Kowa Company, Ltd., Tokyo 103-8433, Japan; suganami@kowa.co.jp; 8 Division of Endocrinology and Metabolism, Department of Medicine, Jichi Medical University, Tochigi 329-0498, Japan; ishibash@jichi.ac.jp

**Keywords:** high-density lipoprotein cholesterol, K-877, pemafibrate, renal dysfunction, safety, selective PPARα modulator, triglyceride

## Abstract

Pemafibrate (K-877) is a novel selective peroxisome proliferator-activated receptor-α modulator (SPPARMα) with a favorable benefit-risk balance. Previous clinical trials of pemafibrate used stringent exclusion criteria related to renal functions. Therefore, we investigated its safety and efficacy in a broader range of patients, including those with chronic kidney disease (CKD). In this multicenter, single-arm, open-label, phase III trial, 0.2–0.4 mg/day pemafibrate was administered for 52 weeks to 189 patients with hypertriglyceridemia and an estimated glomerular filtration rate (eGFR) ≥ 45 mL/min/1.73 m^2^ on statin or regardless of eGFR when statin was not administered. Post-hoc analyses were performed on subgroups stratified by baseline eGFR. Triglyceride levels decreased by 45.9% at week 52 (last-observation-carried-forward). These reductions were not correlated with baseline eGFR. The eGFR < 30 mL/min/1.73 m^2^ subgroup showed the greatest reduction in chylomicron, very low-density lipoprotein, small low-density lipoprotein cholesterol levels, and an increase in high-density lipoprotein cholesterol levels. The incidences of adverse events and adverse drug reactions were 82.0% and 31.7%, respectively, and these were not associated with baseline eGFR. In CKD patients, pemafibrate blood concentrations were not elevated. Pemafibrate showed a good safety profile and efficacy in correcting lipid abnormalities in a broad range of patients, including those with CKD.

## 1. Introduction

Atherosclerotic cardiovascular disease (ASCVD) is a leading cause of death [1,2]. Reducing low-density lipoprotein (LDL) cholesterol (LDL-C) levels is an established pharmacotherapy for ASCVD prevention, and statins are preferred for this purpose. Residual lipid abnormalities, such as elevated triglyceride (TG) and/or reduced high-density lipoprotein (HDL)-cholesterol (HDL-C) levels, are secondary targets [3,4].

Reduced renal function is associated with elevated risks of cardiovascular disease (CVD) and death [5,6]. Atherogenic dyslipidemia, characterized by increased TG-rich lipoproteins and decreased HDL-C levels, is frequently observed in patients with reduced renal function, which itself is associated with CVD risk [7]. Although atherogenic dyslipidemia can be ameliorated with peroxisome proliferator-activated receptor α (PPARα) agonists [8], clinical trials have shown that these agents increase serum creatinine levels [9,10,11,12,13,14]. This adverse reaction may require a dose reduction or treatment discontinuation in patients with reduced renal function [15,16,17]. Additionally, statins and PPARα activators may increase the risk of developing myopathy and rhabdomyolysis, which is greater in patients with chronic kidney disease (CKD) [15,18,19]. Therefore, the concomitant use of these drugs should be avoided in these patients, whereas the risks vary with different fibrates and statins used in combination [20].

Pemafibrate (K-877) is a novel selective PPARα modulator (SPPARMα). It was designed to have a favorable benefit-risk balance and is now approved for the treatment of hyperlipidemia in Japan [21,22,23,24,25,26,27,28,29]. Compared to other PPARα agonists, pemafibrate does not significantly increase, and may even decrease, alanine aminotransferase (ALT) or γ-glutamyltransferase (γ-GT) levels [22,24,26]. Moreover, unlike many other PPARα agonists, pemafibrate is principally excreted via the liver [15,27,30] and exposure to pemafibrate is not dependent on the severity of renal dysfunction [27,31].

The present study is a long-term (52-week) phase III trial of pemafibrate involving patients with an unprecedented broad range of characteristics. Unlike previous trials, this study used less stringent exclusion criteria related to hepatic and renal functions. The main data and results of the post-hoc subgroup analyses on the relative efficacy and safety of pemafibrate with respect to the baseline estimated glomerular filtration rate (eGFR) are presented. The latter analyses were performed because a significant number of patients with renal impairment were enrolled in this study.

## 2. Results

### 2.1. Patients

**Figure 1:**
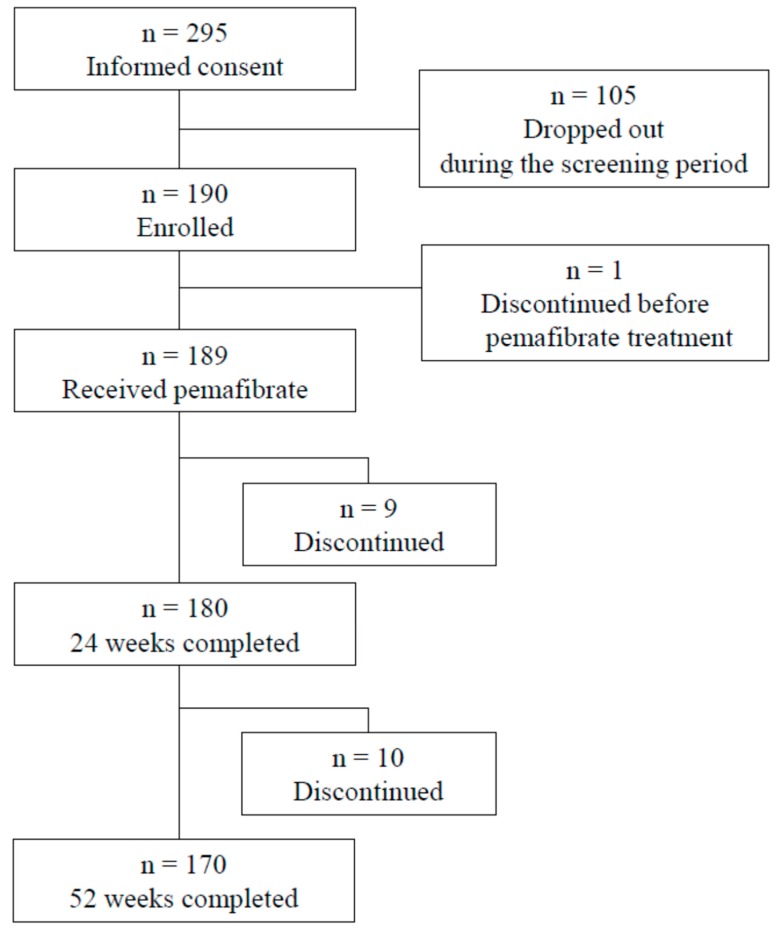
Flow chart of included and excluded patients.

Of the 295 patients who provided written informed consent, 189 received pemafibrate, 105 dropped out during the screening period, and one discontinued use due to an adverse event (AE) occurring before pemafibrate treatment (Figure 1). The baseline patient characteristics are presented in Table 1. The age of the patients was 57.8 ± 10.5 years, and 77.8% were men. Their body mass index (BMI) was 26.0 ± 3.5 kg/m^2^, and 37.0% of the patients had type 2 diabetes mellitus, 53.4% had hypertension, 74.1% had fatty liver, and 57.1% received concomitant statin treatment. TG, HDL-C, and HbA1c levels were 2.82 ± 0.88 mmol/L, 1.18 ± 0.27 mmol/L, and 6.3 ± 0.9%, respectively. In the baseline eGFR subgroups, 21, 123, 34, and eight patients were in the G1, G2, G3a–G3b, and G4–G5 groups, respectively (see “Subjects and Methods” section). Three other patients underwent hemodialysis. The mean age of the patients in the G3a–G3b and G4–G5 subgroups was 12 years older than that of the patients in the G1 group. There were relatively higher rates of hypertension among the patients in the lower eGFR categories. There was no clear correlation between baseline TG or LDL-C and baseline eGFR. The lowest baseline HDL-C, 0.87 ± 0.22 mmol/L, was found in the G4–G5 subgroup.

### 2.2. Efficacy

**Figure 2:**
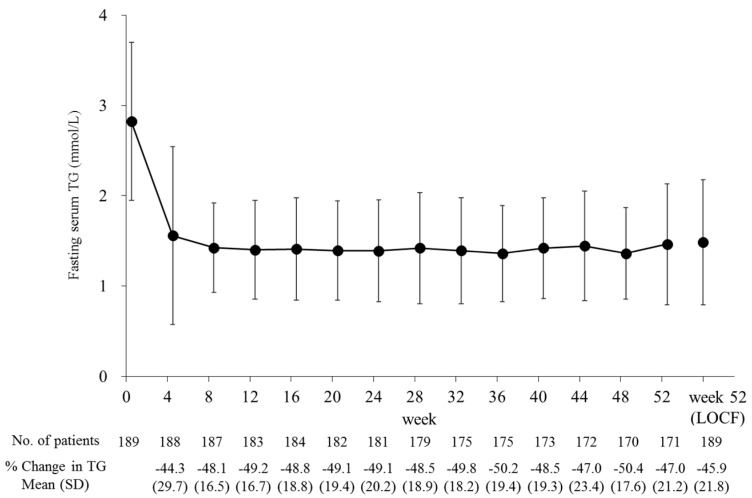
Time course of fasting serum TG levels. Data are presented as means ± SD. TG, triglyceride; LOCF, last-observation-carried-forward; SD, standard deviation.

**Figure 3:**
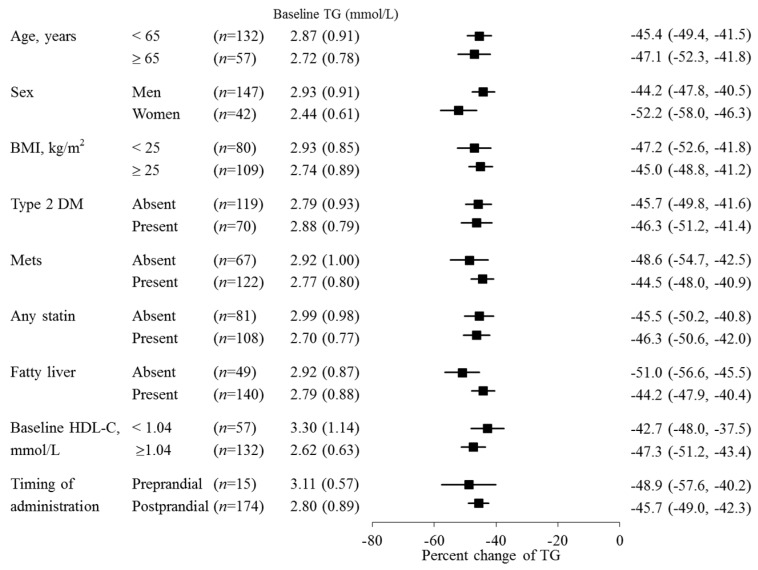
Subgroup analysis for percent change of TG from baseline at week 52 (LOCF). Data of percent change of TG are presented as means (95% CI), and data of baseline TG are presented as means (SD). TG, triglyceride; LOCF, last-observation-carried-forward; SD, standard deviation; CI, confidence interval; BMI, body mass index; DM, diabetes mellitus; Mets, metabolic syndrome.

**Figure 4:**
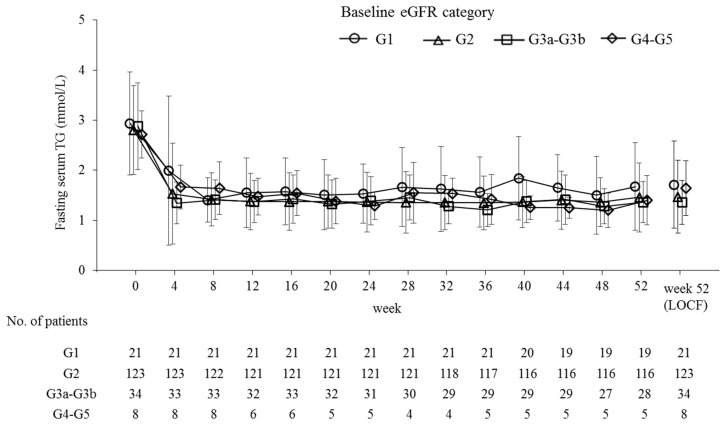
Time course of fasting serum TG levels by baseline eGFR category. Baseline eGFR categories are as follows: G1, eGFR ≥ 90 mL/min/1.73 m^2^; G2, eGFR ≥ 60 and <90 mL/min/1.73 m^2^; G3a–G3b, eGFR ≥ 30 and <60 mL/min/1.73 m^2^; and G4–G5, eGFR < 30 mL/min/1.73 m^2^. Data are presented as means ± standard deviation. TG, triglyceride; eGFR, estimated glomerular filtration rate; LOCF, last-observation-carried-forward.

Fasting serum TG levels decreased by 45.9% from baseline to the last observation (*p* < 0.001) and significantly decreased from baseline over the 52 weeks at each time point (*p* < 0.001; Figure 2). During that time, the pemafibrate dose was increased from 0.2 mg/day to 0.4 mg/day in 29 patients. This adjustment further reduced the TG levels in 17 patients (58.6%). There were no large differences in percent TG reduction either among the prespecified subgroups (Figure 3) or the subgroups categorized by baseline eGFR (Figure 4 and Table 2). The TG reduction in three patients receiving hemodialysis was comparable to that in the total population: Case 1, from 2.62 to 1.39 mmol/L (−46.9%); Case 2, from 3.04 to 1.72 mmol/L (−43.4%); Case 3, from 1.93 to 0.82 mmol/L (−57.5%) during the 52-week treatment.

Pemafibrate treatment significantly increased HDL-C, apolipoprotein (apo) A1, and apoA2 levels, and significantly reduced TG/HDL-C, non-HDL-C, remnant lipoprotein cholesterol (RemL-C), apoB, apoB48, apoC3, and interleukin-1β (IL-1β) levels (Table 2). There were no significant changes in LDL-C levels. High-performance liquid chromatography (HPLC) analysis revealed that the cholesterol content significantly decreased in TG-rich lipoproteins such as chylomicrons (CMs) and very-low-density lipoproteins (VLDLs), and in small to very small LDL particles. In contrast, the cholesterol content significantly increased in medium to very small HDL particles (Table 3). In subgroups with lower baseline eGFR, there were relatively large increases in apoA1, apoA2, HDL-C, medium HDL-C, and small HDL-C, and large decreases in CM-cholesterol (CM-C), VLDL-cholesterol (VLDL-C), small LDL-C, and very small LDL-C levels (Table 2 and Table 3).

### 2.3. Safety

The incidence of total AEs and adverse drug reactions (ADRs) over 52 weeks was 82.0% and 31.7%, respectively (Table 4). AEs with an incidence ≥5% included nasopharyngitis (28.0%) and cholelithiasis (5.8%). The incidence of AEs during weeks 0–12, 12–24, 24–36, 36–48, and 48–52 was 40.2% (76/189), 46.5% (86/185), 39.2% (71/181), 34.7% (60/173), and 12.9% (22/171) of the patients, respectively, showing no increase over time. The incidence of total AEs/ADRs was similar across the baseline eGFR subgroups (Table 4).

Serious adverse events (SAEs) occurred in 8.5% (16/189) of the patients (Table 4 and Table 5). Death due to acute myocardial infarction occurred in one patient in the G2 subgroup. A causal relationship between SAEs and pemafibrate treatment was ruled out in all cases except for cerebral infarction in one patient in the G2 subgroup. All those who underwent hemodialysis experienced SAEs, including malaise in one patient and shunt occlusion/stenosis in two others, of which one also experienced cataracts and upper respiratory tract inflammation. AEs leading to discontinuation occurred in 5.8% (11/189) of the patients (Table 4 and Table 5) and the incidence rate was highest in the G4–G5 subset of this group [37.5% (3/8)]. Relevant events included aortic aneurysm/dissection and carotid artery dissection, chronic kidney disease, and drug eruption in three different patients.

**Figure 5:**
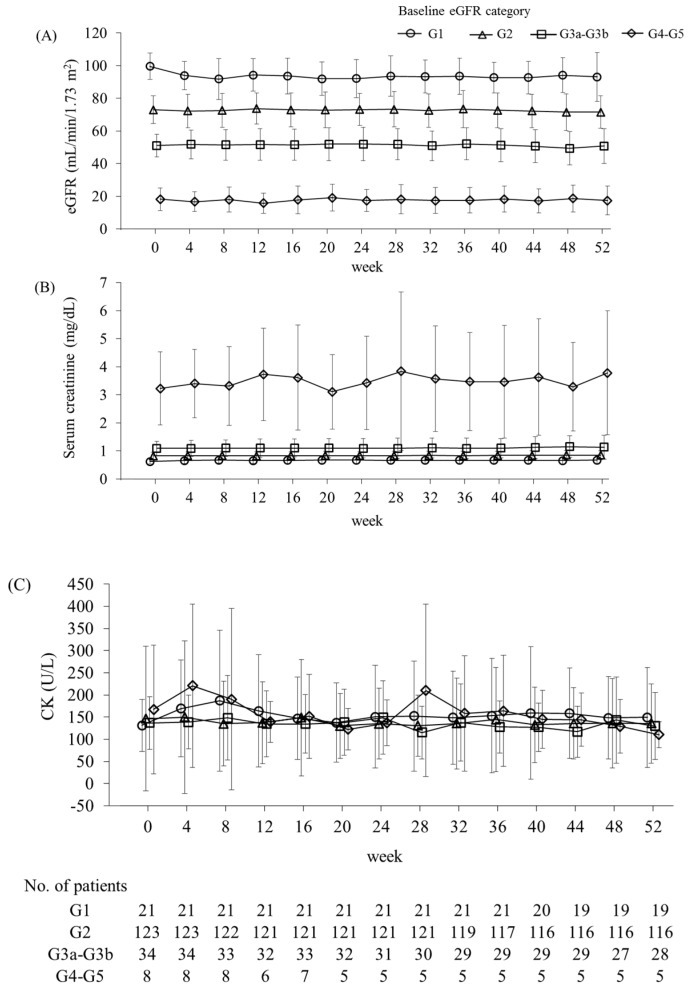
Time course of the levels of eGFR (**A**), serum creatinine (**B**), and CK (**C**) by baseline eGFR category. Baseline eGFR categories are as follows: G1, eGFR ≥90 mL/min/1.73 m^2^; G2, eGFR ≥60 and <90 mL/min/1.73 m^2^; G3a–G3b, eGFR ≥30 and <60 mL/min/1.73 m^2^; and G4–G5, eGFR <30 mL/min/1.73 m^2^. Data are presented as means ± SD. eGFR, estimated glomerular filtration rate; CK, creatine kinase; SD, standard deviation.

No rhabdomyolysis was observed. Myalgia was reported in 2.6% (5/189) of the patients. Four of these patients were in the G2 subgroup and one was in the G3a–G3b subgroup, where three patients and one patient received concomitant statin therapy in each group, respectively. There were no significant changes in creatine kinase (CK) levels over time in any eGFR subgroup (Figure 5 and Table 6). Increases in CK to >2.5 × ULN (upper limit of normal) occurred in 5.8% (11/189) of the patients, and this was evenly distributed across the eGFR subgroups (Table 4), where two patients with baseline eGFR <60 mL/min/1.73 m^2^ and four out of nine patients with baseline eGFR ≥ 60 mL/min/1.73 m^2^ were not taking statins. Among them, one patient who was not on statin therapy in the G2 subgroup increased CK to >5 × ULN. Specifically, the CK level of this patient increased from 215 U/L at baseline to 1452 U/L at week 4 and then decreased to 156 U/L at week 8 without discontinuing pemafibrate treatment. No additional significant changes in CK level were noted until week 52. There were no major changes in eGFR over time in any eGFR subgroup except for a decrease from 99.6 mL/min/1.73 m^2^ at baseline to 91.8 mL/min/1.73 m^2^ after 8 weeks in the G1 group (Figure 5). No patients presented increases in serum creatinine >2× the baseline value (Table 4). γ-GT and alkaline phosphatase (ALP) levels significantly decreased in the G1, G2, and G3a–G3b groups (Table 6).

### 2.4. Plasma Concentrations

**Figure 6:**
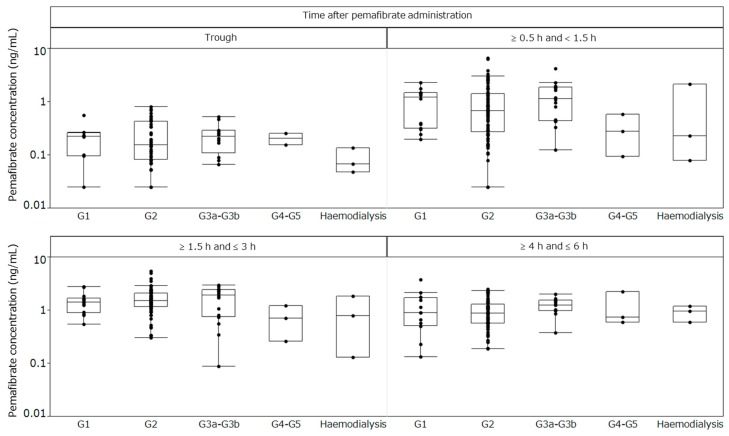
Plasma concentrations of pemafibrate after repeated-dose administration. Baseline eGFR categories are as follows: G1, eGFR ≥ 90 mL/min/1.73 m^2^; G2, eGFR ≥ 60 and <90 mL/min/1.73 m^2^; G3a–G3b, eGFR ≥ 30 and <60 mL/min/1.73 m^2^; G4–G5, eGFR < 30 mL/min/1.73 m^2^. eGFR, estimated glomerular filtration rate.

Plasma concentrations of pemafibrate were obtained from 114 patients among the 189 participants; one had only the trough value. Blood sampling after pemafibrate administration was conducted at week 4 in 23 patients, week 8 in 38 patients, week 12 in 21 patients, week 16 in 14 patients, week 20 in 12 patients, and week 24 in five patients, with the largest number of blood samples collected during week 8. At every trough sampling time and at 0.5–<1.5 h, 1.5–3 h, and 4–6 h after pemafibrate administration, the pemafibrate plasma concentrations were comparable across the subgroups stratified by renal function (Figure 6).

## 3. Discussion

In the present phase III trial, a broad range of patients—including those with impaired renal function (CKD stages G3a, G3b, G4, and G5, and receiving hemodialysis)—was recruited, and the duration of administration (52 weeks) was longer than that in previous pemafibrate trials. Compared to the baseline, pemafibrate reduced fasting serum TG levels by ~50%. There were no significant differences in TG reduction across the prespecified subgroups stratified by baseline patient characteristics. Pemafibrate uptitration from 0.2 mg/day to 0.4 mg/day further reduced TG levels. There were no time-dependent increases in the incidence of AEs/ADRs during long-term pemafibrate administration. Furthermore, the overall efficacy and safety of pemafibrate were similar across all subgroups stratified by baseline eGFR.

The efficacy of pemafibrate to ameliorate an abnormal lipid profile has been demonstrated in previous clinical studies of up to 24 weeks long and was also observed in the present 52-week trial [22,23,24,25,26]. Patients with impaired renal function often have atherogenic dyslipidemia, including high TG and low HDL-C levels, which occurs as a result of impaired TG-rich lipoprotein catabolism associated with decreased lipoprotein lipase (LPL) activity or impaired HDL maturation caused by reduced lecithin-cholesterol acyltransferase (LCAT) activity [7,32,33,34,35]. In addition, apoA1 and apoA2 levels are decreased in these patients [36]. In the present study, although baseline TG levels were not correlated with baseline eGFR levels, baseline TG-rich lipoprotein cholesterol levels (as indicated by CM-C and VLDL-C) were highest in the G4–G5 subgroup. Pemafibrate achieved the greatest reductions in TG-rich lipoprotein levels in the subgroups with low baseline eGFR. In contrast, pemafibrate caused the largest increase in HDL-C, apoA1, and apoA2 levels in the lowest eGFR subgroup. Pemafibrate treatment also resulted in the greatest decreases in small LDL-C and very small LDL-C particles in the subgroup with the lowest baseline eGFR. Therefore, pemafibrate may have substantial influences on the atherogenic lipoprotein profiles frequently observed in patients with impaired renal function. Clarification of the underlying mechanism of these findings will require further investigation.

The present study had no control group. Therefore, it could not be determined whether the incidences of AEs and ADRs in patients receiving pemafibrate were higher or lower than those receiving other treatments or placebo. According to an earlier report, hypertriglyceridemic patients with normal renal function receiving pemafibrate treatment had lower incidences of AEs and ADRs than those receiving fenofibrate [24,26]. The AE and ADR incidences and profiles in patients administered pemafibrate alone or in combination with a statin were comparable to those observed in the placebo group [22,23,25,26].

The major AEs of concern associated with fibrate administration are rhabdomyolysis/myopathy and renal function decline [15]. The risk of rhabdomyolysis/myopathy may increase when fibrates are administered in combination with statins [20]. However, no cases of rhabdomyolysis were observed in the present study. CK elevation >5 × ULN was observed in only one patient in the G2 subgroup. The incidences of myalgia and CK elevation >2.5 × ULN were not correlated with baseline eGFR. Moreover, none of these AEs led to treatment discontinuation. CK levels did not significantly increase in patients with reduced renal function. However, the number of patients with baseline eGFR < 60 mL/min/1.73 m^2^ in this study was limited; thus, larger-scale trials are required to confirm the safety of pemafibrate in patients with CKD.

Here, pemafibrate treatment was shown to significantly increase serum creatinine levels in the G1 and G2 subgroups with mean changes of 0.06 and 0.02 mg/dL, respectively. These increases are comparable to those observed in previous studies but are lower than those reported for fenofibrate [22,23,24,25,26]. Furthermore, there were no significant changes in eGFR in the G3a–G3b and G4–G5 subgroups.

In this study, we treated three patients undergoing hemodialysis for 52 weeks. In hemodialysis patients, LDL-C levels are not high, but lipid abnormalities such as high TG, high small LDL particles, and low HDL-C may occur [7]. Conventional fibrates are contraindicated in patients with severe renal dysfunction [37]. However, several reports have evaluated the efficacy and safety of fibrates in patients receiving dialysis [38,39,40]. In the present study, the TG reductions in the three patients receiving hemodialysis were comparable to those in the total population, and although AEs occurred in all three patients on hemodialysis, none of these AEs were deemed to be related to pemafibrate treatment. However, pemafibrate efficacy and safety data are limited for hemodialysis patients; therefore, further studies are necessary to validate the usefulness of pemafibrate administration in hemodialysis patients.

The patient who died of an acute myocardial infarction was a smoker and had undergone percutaneous coronary intervention before participating in the present study. Therefore, the investigator concluded that this particular AE was not associated with pemafibrate treatment. A causal relationship between pemafibrate and one cerebral infarction event could not be excluded by the investigator. This patient had hypertension, fatty liver, and type 2 diabetes mellitus, and smoked ~20 cigarettes/day. Consequently, this patient was most likely already at increased risk of cerebral infarction.

After oral administration of ^14^C-pemafibrate, pemafibrate was mainly excreted into the bile with very little urinary excretion of unchanged pemafibrate (<0.5%) [27,30]. Moreover, a pharmacokinetic study of a single oral administration of pemafibrate in patients with normal and impaired renal function showed no increase in systemic exposure in a renal function-dependent manner [27,31]. Consistent with these findings, the present study showed no increase in the plasma concentration of pemafibrate in patients with severely impaired renal function or those on dialysis compared with that in patients with normal renal function, even in the steady state after repeated administration. Although patients with impaired renal function frequently have comorbidities such as elevated TG and low HDL-C levels, precautions are required for the use of conventional fibrate drugs because they are primarily excreted via the kidneys, and their plasma concentrations increase in patients with impaired renal function [41,42,43,44,45,46]. Therefore, pemafibrate may offer a new therapeutic option for dyslipidemic patients with impaired renal function.

The limitations of the present study are as follows: (1) this study had no control group, and the presence of impaired renal function by itself may have been associated with a higher occurrence of AEs. Therefore, a randomized, placebo-controlled trial is necessary to assess pemafibrate safety in patients with impaired renal function; (2) the number of patients in the G4–G5 subgroup was limited. A larger-scale study with enriched enrollment of such patients is needed to confirm pemafibrate efficacy and safety in this population; (3) the study participants were all Japanese. For this reason, it is unknown whether similar results would be found in other ethnic populations. In the U.S., a pemafibrate trial is currently underway involving patients with extremely high TG levels and mildly to moderately impaired renal function (NCT03011450).

## 4. Subjects and Methods

This was a multicenter, single-arm, open-label, phase III trial. It was conducted at 32 sites in Japan between May 2014 and November 2015. Written informed consent was obtained from the participants before trial initiation. The protocol was approved by the institutional review board. This trial was conducted in accordance with the principles of the Declaration of Helsinki and the “Ministerial Ordinance on Good Clinical Practice” (GCP Ordinance) and was registered with the Japan Pharmaceutical Information Center (JAPIC) (JapicCTI-142496, 2 April 2014).

The inclusion criteria were as follows: (1) patients with dyslipidemia aged ≥20 years at the time of informed consent; (2) men and postmenopausal women; (3) fasting serum TG ≥1.70 mmol/L (150 mg/dL) at two consecutive measurements during screening; and (4) patients who followed dietary and physical exercise guidance for ≥12 weeks before enrollment.

The major exclusion criteria were as follows: (1) patients with fasting serum TG ≥5.65 mmol/L (500 mg/dL) during screening; (2) patients with poorly controlled diabetes mellitus (hemoglobin A1c [HbA1c] ≥ 10.5%); (3) patients with concurrent poorly controlled thyroid disease; (4) men with serum creatinine ≥2.5 mg/dL and women with serum creatinine ≥2.0 mg/dL during screening who were already on statin therapy; (5) patients with eGFR <45 mL/min/1.73 m^2^ during screening who were already on statin therapy; (6) patients with CK >5×ULN (270 IU/L for men, 150 IU/L for women) during screening who were already on statin therapy; (7) patients with serious liver disease (cirrhosis Child–Pugh Class B or higher); (8) patients with gallstones or serious biliary tract disease; (9) patients who had suffered an acute myocardial infarction or stroke within three months before informed consent; and (10) patients with New York Heart Association class III or IV heart failure. During the trial period, the following drugs were prohibited from concomitant use: fibrates, p-glycoprotein inhibitors, breast cancer-resistance protein inhibitors, and organic anion transporting polypeptide 1B1 or 1B3 inhibitors. Initiation, discontinuation, and modification of the dosage regimens of corticosteroids, protease inhibitors, anabolic steroid hormones, progestogens, lipid lowering agents, or hypoglycemic agents other than the abovementioned drugs prohibited for concomitant use were essentially prohibited. However, the addition or dosage increase of lipid lowering agents and hypoglycemic agents was permitted if deemed necessary. The addition of lipid lowering agents and thiazolidinediones was prohibited for the first 12 weeks of the study so that their effects on drug efficacy could be assessed at week 12.

During the screening period (8 weeks prior to treatment initiation), tests were performed twice to determine patient eligibility. Thereafter, eligible patients orally received pemafibrate 0.2 mg/day (twice daily) for 52 weeks. From week 12 of the treatment period onwards, the investigators were instructed that the dose could be increased from 0.2 mg/day to 0.4 mg/day (twice daily) if there was an inadequate response to the initial dose based on fasting serum TG levels ≥1.70 mmol/L (150 mg/dL).

Fasting blood and urine samples were collected at each visit. Blood and urine sample in patients with hemodialysis were collected just before dialysis. LDL-C levels were measured using the direct method. Lipoprotein fractions were measured by HPLC [47] at baseline and weeks 12 and 40. All central measurements were made by LSI Medience Corp. (Chiyoda-ku, Tokyo, Japan) except for the lipoprotein fraction, which was determined by Skylight Biotech Inc. (Akita, Japan).

The primary efficacy endpoint was the percent change in fasting serum TG from baseline to the last evaluation point. The primary safety endpoints were the incidence of an AE or ADR occurring after drug administration during the study. Secondary efficacy endpoints included percent changes in lipid variables and changes in inflammation variables at week 52 [using the last-observation-carried-forward (LOCF)]. Secondary safety endpoints included changes in the levels of aspartate aminotransferase (AST), ALT, γ-GT, ALP, serum creatinine, eGFR, and CK at week 52. Each baseline value was defined as (1) the mean of the corresponding values in the first and second tests at the screening examination and at week 0 of the treatment period for fasting serum TG, HDL-C, total cholesterol (TC), LDL-C, and non-HDL-C; and (2) the value at week 0 of the treatment period for the other secondary variables. Efficacy and safety were established post hoc by subgroups stratified by baseline eGFR as follows: G1 (normal or high; ≥90 mL/min/1.73 m^2^), G2 (mildly decreased; ≥60 and <90 mL/min/1.73 m^2^), G3a–G3b (mildly to moderately decreased and moderately to severely decreased; ≥30 and <60 mL/min/1.73 m^2^), and G4–G5 (severely decreased and kidney failure; <30 mL/min/1.73 m^2^), according to the Kidney Disease Improving Global Outcomes (KDIGO) 2012 Clinical Practice Guideline for the Evaluation and Management of Chronic Kidney Disease [48]. The plasma concentration of pemafibrate was measured with liquid chromatography-tandem mass spectrometry (LC-MS-MS) only at the institutions where this procedure was feasible and in patients who provided informed consent. Blood sampling for trough values was conducted before the morning dose of pemafibrate in parallel with that for fasting blood laboratory tests in weeks 4, 8, and 12 of the treatment period. Blood sampling after pemafibrate administration was carried out once between weeks 4 and 24 at 0.5–1.5, 1.5–3, and 4–6 h after pemafibrate administration.

The target sample size was set to 170 patients based on the number required to evaluate safety according to the International Conference on Harmonisation (ICH)-E1 guideline “Extent of population exposure to assess clinical safety for drugs intended for long-term treatment of non-life-threatening conditions”. For the primary efficacy endpoint, one-sample *t*-tests were performed. The numbers of patients with AEs and ADRs and the incidences of AEs and ADRs were calculated in the analysis of primary safety endpoints. For the secondary efficacy endpoints, one-sample *t*-tests or Wilcoxon signed-rank tests [for high-sensitivity C-reactive protein (hsCRP) and IL-1β] were performed. The secondary safety endpoints were analyzed with Wilcoxon signed-rank tests. A two-sided significance level of 0.05 was used. SAS v. 9.3 (SAS Institute Inc., Cary, NC, USA) was used for these analyses. Where indicated, the data are expressed as means ± standard deviation (SD).

## 5. Conclusions

In conclusion, 0.2 to 0.4 mg/day pemafibrate presented a good safety profile and excellent efficacy to treat serum lipid abnormalities in a broad range of patients, including those with CKD.

## Figures and Tables

**Table 1:** Characteristics of patients at baseline.

Parameter	All Participants	Baseline eGFR Category				Hemodialysis
		G1	G2	G3a–G3b	G4–G5	
*n*	189	21	123	34	8	3
Age (years)	57.8 (10.5)	52.6 (9.8)	55.9 (9.3)	65.5 (9.7)	64.6 (12.8)	67.7 (12.5)
Sex, Men	147 (77.8)	16 (76.2)	98 (79.7)	25 (73.5)	7 (87.5)	1 (33.3)
BMI (kg/m^2^)	26.0 (3.5)	26.6 (4.6)	26.1 (3.4)	25.8 (2.5)	26.0 (2.3)	23.4 (7.3)
Type 2 diabetes	70 (37.0)	10 (47.6)	39 (31.7)	16 (47.1)	4 (50.0)	1 (33.3)
Hypertension	101 (53.4)	8 (38.1)	57 (46.3)	26 (76.5)	7 (87.5)	3 (100)
Fatty liver	140 (74.1)	19 (90.5)	91 (74.0)	26 (76.5)	4 (50.0)	0
Use of a statin	108 (57.1)	11 (52.4)	77 (62.6)	18 (52.9)	2 (25.0)	0
TG (mmol/L)	2.82 (0.88)	2.94 (1.03)	2.80 (0.89)	2.88 (0.86)	2.72 (0.47)	2.47 (0.53)
HDL-C (mmol/L)	1.18 (0.27)	1.24 (0.36)	1.19 (0.24)	1.19 (0.31)	0.87 (0.22)	1.12 (0.42)
LDL-C (mmol/L)	3.09 (0.82)	3.20 (0.88)	3.08 (0.78)	2.96 (0.72)	3.42 (1.18)	3.45 (2.09)
Serum creatinine (mg/dL)	1.05 (1.00)	0.62 (0.10)	0.82 (0.12)	1.09 (0.25)	3.23 (1.30)	7.35 (1.68)
eGFR (mL/min/1.73 m^2^)	68.6 (20.3)	99.6 (8.0)	72.9 (8.5)	51.1 (7.0)	18.2 (6.9)	5.6 (1.4)
HbA1c (%)	6.3 (0.9)	6.5 (1.1)	6.2 (0.8)	6.6 (1.0)	6.1 (0.4)	6.7 (1.9)

Data are presented as means (SD) for continuous parameters and *n* (%) for categorical parameters. Baseline eGFR categories are as follows: G1, eGFR ≥90 mL/min/1.73 m^2^; G2, eGFR ≥60 and <90 mL/min/1.73 m^2^; G3a–G3b, eGFR ≥30 and <60 mL/min/1.73 m^2^; G4–G5, eGFR <30 mL/min/1.73 m^2^. BMI, body mass index; TG, triglyceride; HDL-C, high-density lipoprotein cholesterol; LDL-C, low-density lipoprotein cholesterol; eGFR, estimated glomerular filtration rate; HbA1c, hemoglobin A1c; SD, standard deviation.

**Table 2:** Changes from baseline in lipid levels and inflammatory parameters during the 52-week treatment period.

Parameter		All Participants	Baseline eGFR Category			
			G1	G2	G3a–G3b	G4–G5
TG (mmol/L)	*n*	189	21	123	34	8
	Baseline	2.82 (0.88)	2.94 (1.03)	2.80 (0.89)	2.88 (0.86)	2.72 (0.47)
	Week 52 (LOCF)	1.48 (0.69)	1.71 (0.87)	1.47 (0.72)	1.36 (0.44)	1.64 (0.54)
	% Change	−45.9 (21.8) ***	−41.4 (23.0) ***	−45.8 (23.1) ***	−51.1 (15.4) ***	−37.7 (23.6) **
HDL-C (mmol/L)	*n*	189	21	123	34	8
	Baseline	1.18 (0.27)	1.24 (0.36)	1.19 (0.24)	1.19 (0.31)	0.87 (0.22)
	Week 52 (LOCF)	1.33 (0.34)	1.29 (0.44)	1.34 (0.32)	1.38 (0.35)	1.18 (0.37)
	% Change	13.1 (17.1) ***	3.6 (16.9)	11.9 (16.2) ***	17.0 (13.0) ***	34.1 (23.7) **
TG/HDL-C [(mmol/L)/(mmol/L)]	*n*	189	21	123	34	8
	Baseline	2.58 (1.22)	2.69 (1.52)	2.49 (1.13)	2.69 (1.37)	3.30 (0.95)
	Week 52 (LOCF)	1.27 (0.85)	1.58 (1.03)	1.24 (0.87)	1.09 (0.55)	1.68 (1.12)
	% Change	−49.7 (25.7) ***	−40.5 (33.2) ***	−48.9 (26.5) ***	−57.0 (15.9) ***	−51.0 (22.4) ***
LDL-C (mmol/L)	*n*	189	21	123	34	8
	Baseline	3.09 (0.82)	3.20 (0.88)	3.08 (0.78)	2.96 (0.72)	3.42 (1.18)
	Week 52 (LOCF)	3.02 (0.75)	3.12 (0.89)	3.03 (0.76)	2.97 (0.62)	2.82 (0.85)
	% Change	2.2 (30.4)	2.7 (33.7)	2.1 (29.2)	5.1 (30.0)	−8.8 (41.2)
non HDL-C (mmol/L)	*n*	189	21	123	34	8
	Baseline	4.03 (0.79)	4.18 (0.87)	4.00 (0.73)	3.92 (0.61)	4.54 (1.34)
	Week 52 (LOCF)	3.63 (0.83)	3.77 (1.01)	3.64 (0.85)	3.56 (0.66)	3.49 (0.96)
	% Change	−8.7 (18.8) ***	−8.3 (21.1)	−8.4 (17.4) ***	−7.7 (18.0) *	−17.3 (33.5)
RemL-C (mmol/L)	*n*	187	21	122	34	7
	Baseline	0.48 (0.26)	0.54 (0.30)	0.46 (0.26)	0.48 (0.26)	0.59 (0.15)
	Week 52 (LOCF)	0.18 (0.13)	0.21 (0.15)	0.18 (0.14)	0.17 (0.09)	0.24 (0.17)
	% Change	−57.2 (28.7) ***	−59.9 (21.1) ***	−56.0 (31.8) ***	−59.3 (22.9) ***	−57.1 (25.8) **
apoA1 (mg/dL)	*n*	187	21	122	34	7
	Baseline	131.6 (19.8)	134.2 (22.0)	132.2 (18.2)	133.5 (20.0)	105.4 (19.7)
	Week 52 (LOCF)	137.6 (22.4)	133.8 (26.0)	137.2 (20.0)	144.0 (25.5)	122.4 (20.8)
	% Change	5.0 (11.2) ***	0.0 (13.9)	4.1 (10.5) ***	8.0 (10.0) ***	16.7 (10.6) **
apoA2 (mg/dL)	*n*	187	21	122	34	7
	Baseline	30.3 (4.5)	31.4 (5.1)	30.8 (4.2)	29.5 (4.7)	24.2 (3.3)
	Week 52 (LOCF)	38.2 (6.9)	37.9 (5.9)	38.7 (6.8)	37.8 (6.8)	32.8 (7.1)
	% Change	27.0 (19.5) ***	22.2 (18.4) ***	26.3 (17.6) ***	29.4 (22.7) ***	35.3 (21.7) **
apoB (mg/dL)	*n*	187	21	122	34	7
	Baseline	93.6 (16.8)	95.6 (17.8)	93.4 (16.5)	91.9 (13.8)	97.4 (23.5)
	Week 52 (LOCF)	88.1 (18.6)	93.0 (23.1)	88.0 (18.5)	87.9 (15.4)	76.1 (21.6)
	% Change	−4.5 (19.9) **	−1.9 (19.6)	−4.5 (19.6) *	−3.0 (17.7)	−18.1 (29.5)
apoB48 (μg/mL)	*n*	188	21	123	34	7
	Baseline	9.1 (5.8)	10.3 (6.7)	8.5 (5.4)	9.1 (5.6)	12.8 (4.1)
	Week 52 (LOCF)	3.8 (3.0)	3.7 (2.1)	3.3 (2.8)	4.0 (2.1)	7.8 (4.1)
	% Change	−53.0 (30.9) ***	−60.4 (21.5) ***	−54.4 (33.0) ***	−48.1 (26.6) ***	−34.9 (34.4) *
apoC3 (mg/dL)	*n*	187	21	122	34	7
	Baseline	15.1 (4.5)	15.9 (5.0)	14.7 (4.3)	15.9 (5.2)	13.2 (1.1)
	Week 52 (LOCF)	9.8 (3.2)	10.6 (3.8)	9.3 (3.1)	10.5 (2.5)	10.5 (2.9)
	% Change	−32.4 (19.6) ***	−32.4 (15.3) ***	−34.2 (20.1) ***	−30.0 (18.3) ***	−20.2 (22.6)
hsCRP (mg/dL)	*n*	186	21	123	33	6
	Baseline	0.11 (0.20)	0.18 (0.43)	0.09 (0.11)	0.13 (0.19)	0.23 (0.39)
	Week 52 (LOCF)	0.13 (0.26)	0.23 (0.58)	0.12 (0.19)	0.13 (0.15)	0.06 (0.03)
	Change	0.02 (0.30)	0.04 (0.71)	0.03 (0.17)	−0.00 (0.22)	−0.17 (0.37)
IL-1β (pg/mL)	*n*	183	21	123	31	5
	Baseline	0.16 (0.22)	0.21 (0.28)	0.15 (0.20)	0.17 (0.28)	0.13 (0.10)
	Week 52 (LOCF)	0.09 (0.08)	0.08 (0.08)	0.08 (0.08)	0.11 (0.10)	0.13 (0.12)
	Change	−0.07 (0.24) ***	−0.13 (0.31) *	−0.06 (0.22) ***	−0.06 (0.30)	−0.00 (0.17)

Data are presented as means (SD). Baseline eGFR categories are as follows: G1, eGFR ≥ 90 mL/min/1.73 m^2^; G2, eGFR ≥ 60 and <90 mL/min/1.73 m^2^; G3a–G3b, eGFR ≥ 30 and <60 mL/min/1.73 m^2^; G4–G5, eGFR < 30 mL/min/1.73 m^2^. Three patients in hemodialysis were included in the all participants category but were excluded from the G4–G5 category. * *p* < 0.05, ** *p* < 0.01, *** *p* < 0.001 vs. baseline by Wilcoxon signed-rank tests for hsCRP and IL-1β, and by one sample *t*-tests for all others. LOCF, last-observation-carried-forward; eGFR, estimated glomerular filtration rate; TG, triglyceride; HDL-C, high-density lipoprotein cholesterol; LDL-C, low-density lipoprotein cholesterol; RemL-C, remnant lipoprotein cholesterol; Apo, apolipoprotein; hsCRP, high-sensitivity C-reactive protein; IL-1β, interleukin 1β; SD, standard deviation.

**Table 3:** Changes from baseline in the level of cholesterol in CM, VLDL, LDL, and HDL subclasses measured by HPLC.

Parameter		All Participants	Baseline eGFR Category			
			G1	G2	G3a–G3b	G4–G5
(mmol/L)	*n*	188	21	123	34	7
CM-C	Baseline	0.154 (0.135)	0.158 (0.114)	0.151 (0.146)	0.156 (0.121)	0.188 (0.082)
	Week 52 (LOCF)	0.039 (0.038)	0.064 (0.055)	0.035 (0.036)	0.035 (0.025)	0.039 (0.037)
	% Change	−64.3 (35.5) ***	−54.1 (46.0) ***	−63.9 (36.1) ***	−67.7 (29.1) ***	−79.4 (14.0) ***
VLDL-C	Baseline	1.196 (0.335)	1.260 (0.390)	1.164 (0.300)	1.177 (0.319)	1.559 (0.474)
	Week 52 (LOCF)	0.866 (0.264)	0.932 (0.323)	0.834 (0.234)	0.892 (0.293)	1.076 (0.372)
	% Change	−24.9 (22.5) ***	−24.6 (20.0) ***	−25.3 (22.9) ***	−22.9 (21.5) ***	−28.7 (25.5) *
Large LDL-C	Baseline	0.504 (0.185)	0.529 (0.186)	0.498 (0.175)	0.489 (0.182)	0.570 (0.279)
	Week 52 (LOCF)	0.748 (0.183)	0.724 (0.130)	0.765 (0.200)	0.720 (0.150)	0.689 (0.143)
	% Change	63.1 (63.2) ***	54.5 (62.1) ***	67.7 (68.1) ***	59.1(44.2) ***	39.9 (61.3)
Medium LDL-C	Baseline	1.102 (0.330)	1.134 (0.325)	1.120 (0.337)	1.030 (0.295)	1.058 (0.382)
	Week 52 (LOCF)	1.194 (0.300)	1.244 (0.349)	1.226 (0.307)	1.127 (0.208)	0.920 (0.248)
	% Change	17.3 (49.9) ***	15.5 (37.9)	18.7 (53.7) ***	18.9 (45.1) *	−2.8 (44.2)
Small LDL-C	Baseline	0.682 (0.190)	0.711 (0.236)	0.690 (0.187)	0.650 (0.160)	0.643 (0.223)
	Week 52 (LOCF)	0.517 (0.165)	0.576 (0.223)	0.522 (0.158)	0.501 (0.139)	0.381 (0.154)
	% Change	−20.6 (28.6) ***	−18.0 (21.3) ***	−20.6 (29.3) ***	−18.9 (28.2) ***	−38.6 (23.9) **
Very small LDL-C	Baseline	0.254 (0.080)	0.262 (0.064)	0.250 (0.080)	0.264 (0.084)	0.258 (0.113)
	Week 52 (LOCF)	0.210 (0.061)	0.233 (0.080)	0.211 (0.056)	0.207 (0.063)	0.158 (0.056)
	% Change	−11.6 (30.2) ***	−10.3 (23.2)	−9.7 (30.1) ***	−15.8 (29.0) **	−33.4 (21.4) **
Very large HDL-C	Baseline	0.046 (0.015)	0.047 (0.016)	0.046 (0.013)	0.048 (0.016)	0.038 (0.015)
	Week 52 (LOCF)	0.048 (0.016)	0.047 (0.018)	0.048 (0.016)	0.051 (0.017)	0.044 (0.020)
	% Change	4.4 (21.5) **	0.8 (18.3)	4.1 (21.0) *	5.9 (20.4)	17.1 (36.1)
Large HDL-C	Baseline	0.130 (0.083)	0.123 (0.103)	0.130 (0.080)	0.139 (0.082)	0.096 (0.085)
	Week 52 (LOCF)	0.115 (0.088)	0.107 (0.100)	0.115 (0.088)	0.124 (0.082)	0.096 (0.090)
	% Change	−9.2 (45.1) **	−14.1 (43.2)	−10.9 (44.7) **	−4.9 (42.6)	8.2 (53.4)
Medium HDL-C	Baseline	0.374 (0.114)	0.389 (0.131)	0.382 (0.105)	0.372 (0.118)	0.216 (0.072)
	Week 52 (LOCF)	0.443 (0.155)	0.441 (0.187)	0.448 (0.146)	0.447 (0.170)	0.336 (0.141)
	% Change	19.3 (24.4) ***	11.4 (21.3) *	17.7 (23.9) ***	20.6 (22.3) ***	53.2 (25.3) **
Small HDL-C	Baseline	0.388 (0.076)	0.414 (0.092)	0.393 (0.070)	0.382 (0.072)	0.281 (0.057)
	Week 52 (LOCF)	0.473 (0.087)	0.482 (0.097)	0.477 (0.079)	0.467 (0.082)	0.395 (0.106)
	% Change	24.2 (23.3) ***	18.1 (16.8) ***	23.6 (23.3) ***	24.6 (22.4) ***	39.1 (20.9) **
Very small HDL-C	Baseline	0.148 (0.032)	0.158 (0.032)	0.146 (0.034)	0.154 (0.023)	0.128 (0.035)
	Week 52 (LOCF)	0.181 (0.033)	0.186 (0.035)	0.181 (0.031)	0.180 (0.024)	0.155 (0.042)
	% Change	27.9 (36.6) ***	20.6 (25.0) **	30.9 (39.7) ***	19.7 (22.8) ***	23.5 (25.9)

Data are presented as means (SD). Lipoprotein fractions were measured by HPLC at baseline and at week 12 and week 40. Baseline eGFR categories are as follows: G1, eGFR ≥90 mL/min/1.73 m^2^; G2, eGFR ≥60 and <90 mL/min/1.73 m^2^; G3a–G3b, eGFR ≥30 and <60 mL/min/1.73 m^2^; G4–G5, eGFR <30 mL/min/1.73 m^2^. Three patients in hemodialysis were included in the all participants category but were excluded from the G4–G5 category. * *p* < 0.05, ** *p* < 0.01, *** *p* < 0.001 vs. baseline by one sample *t*-test. eGFR, estimated glomerular filtration rate; CM-C, chylomicron cholesterol; VLDL-C, very-low-density lipoprotein cholesterol; LDL-C, low-density lipoprotein cholesterol; HDL-C, high-density lipoprotein cholesterol; HPLC, high-performance liquid chromatography; LOCF, last-observation-carried-forward; SD, standard deviation.

**Table 4:** Summary of AEs and ADRs.

Parameter	All Participants	Baseline eGFR Category				Hemodialysis
		G1	G2	G3a–G3b	G4–G5	
*n*	189	21	123	34	8	3
AE						
Total	155 (82.0)	17 (81.0)	97 (78.9)	31 (91.2)	7 (87.5)	3 (100)
Serious	16 (8.5)	2 (9.5)	4 (3.3)	5 (14.7)	2 (25.0)	3 (100)
Leading to withdrawal	11 (5.8)	2 (9.5)	3 (2.4)	3 (8.8)	3 (37.5)	0
ADR						
Total	60 (31.7)	9 (42.9)	34 (27.6)	15 (44.1)	2 (25.0)	0
Serious	1 (0.5)	0	1 (0.8)	0	0	0
Leading to withdrawal	7 (3.7)	2 (9.5)	2 (1.6)	1 (2.9)	2 (25.0)	0
CK > ULN × 2.5	11 (5.8)	1 (4.8)	8 (6.5)	1 (2.9)	1 (12.5)	0
CK > ULN × 5	1 (0.5)	0	1 (0.8)	0	0	0
sCr > Baseline × 2.0	0	0	0	0	0	0

Data are presented as the number of patients (%). Baseline eGFR categories are as follows: G1, eGFR ≥90 mL/min/1.73 m^2^; G2, eGFR ≥60 and <90 mL/min/1.73 m^2^; G3a–G3b, eGFR ≥30 and <60 mL/min/1.73 m^2^; G4–G5, eGFR <30 mL/min/1.73 m^2^. eGFR, estimated glomerular filtration rate; AE, adverse event; ADR, adverse drug reaction; CK, creatine kinase; ULN, upper limit of normal; sCr, serum creatinine.

**Table 5:** Preferred terms of AEs (underlined events are ADRs).

AEs/ADRs	All	Baseline eGFR Category								Hemodialysis	
		G1		G2		G3a–G3b		G4–G5			
*n*	189	21		123		34		8		3	
Serious	16	2		4		5		2		3	
		G1-1	Diabetes mellitus	G2-1	Inguinal hernia Cryptorchism	G3-1	Aortic aneurysm	G45-1	Gastric adenoma Adenocarcinoma gastric	H-1	Shunt occlusion Shunt stenosis
		G1-2	Myocardial ischemia	G2-2	Sepsis	G3-2	Adenocarcinoma gastric	G45-2	Spinal compression fracture Aortic aneurysm Aortic dissection Carotid artery dissection	H-2	Malaise
				G2-3	Cerebral infarction	G3-3	Pneumonia			H-3	Shunt stenosis Cataract Upper respiratory tract inflammation
				G2-4	Acute myocardial infarction	G3-4	Pneumonia				
						G3-5	Myocardial ischemia				
Leading to withdrawal	11	2		3		3		3		0	
		G1-3	Cholelithiasis	G2-5	AST increased ALT increased	G3-2	Adenocarcinoma gastric	G45-2	Aortic aneurysm Aortic dissection Carotid artery dissection		
		G1-4	Diabetes mellitus	G2-6	Cholelithiasis	G3-3	Pneumonia				
				G2-4	Acute myocardial infarction	G3-6	LDL increased Cholelithiasis	G45-3	Chronic kidney disease		
								G45-4	Drug eruption		

Preferred terms are based on MedDRA Ver. 18.0. Baseline eGFR categories are as follows: G1, eGFR ≥90 mL/min/1.73 m^2^; G2, eGFR ≥60 and <90 mL/min/1.73 m^2^; G3a–G3b, eGFR ≥30 and <60 mL/min/1.73 m^2^; G4–G5, eGFR <30 mL/min/1.73 m^2^. AE, adverse event; ADR, adverse drug reaction; eGFR, estimated glomerular filtration rate; LDL, low-density lipoprotein; AST, aspartate aminotransferase; ALT, alanine aminotransferase.

**Table 6:** Changes from baseline in the levels of safety parameters.

Parameter		All Participants	Baseline eGFR Category			
			G1	G2	G3a–G3b	G4–G5
AST (U/L)	*n*	166	18	113	27	5
	Baseline	27.4 (11.3)	26.2 (5.6)	27.7 (11.1)	29.5 (15.0)	18.0 (3.7)
	Week 52	25.8 (12.0)	28.7 (16.2)	26.1 (12.4)	24.7 (6.3)	21.6 (5.9)
	Change	−1.5 (11.3) *	2.5 (14.9)	−1.7 (10.5) *	−4.7 (12.6) *	3.6 (3.0)
ALT (U/L)	*n*	171	19	116	28	5
	Baseline	31.0 (17.7)	29.3 (11.8)	33.3 (19.4)	26.8 (12.5)	18.4 (12.1)
	Week 52	23.0 (16.1)	26.0 (15.0)	24.1 (17.6)	18.5 (7.6)	20.0 (17.6)
	Change	−8.0 (13.7) ***	−3.3 (13.1)	−9.2 (14.6) ***	−8.3 (10.8) ***	1.6 (6.2)
γ-GT (U/L)	*n*	171	19	116	28	5
	Baseline	55.0 (52.3)	73.6 (96.6)	55.2 (41.3)	50.8 (56.3)	22.2 (10.4)
	Week 52	32.2 (35.0)	48.6 (66.2)	32.3 (31.3)	25.0 (16.3)	18.0 (14.2)
	Change	−22.8 (31.7) ***	−24.9 (37.8) **	−22.9 (28.4) ***	−25.8 (42.7) ***	−4.2 (8.2)
ALP (U/L)	*n*	171	19	116	28	5
	Baseline	225.5 (57.0)	227.6 (59.9)	223.1 (58.1)	229.8 (53.9)	237.8 (56.9)
	Week 52	149.5 (44.7)	154.1 (40.0)	150.6 (47.4)	143.3 (36.0)	137.0 (59.5)
	Change	−76.0 (36.3) ***	−73.5 (38.8) ***	−72.5 (32.9) ***	−86.4 (41.3) ***	−100.8 (54.5)
Serum creatinine (mg/dL)	*n*	171	19	116	28	5
	Baseline	1.02 (0.98)	0.62 (0.10)	0.83 (0.13)	1.08 (0.27)	2.98 (1.19)
	Week 52	1.09 (1.23)	0.67 (0.14)	0.85 (0.16)	1.13 (0.43)	3.78 (2.21)
	Change	0.07 (0.34) ***	0.06 (0.09) **	0.02 (0.07) **	0.06 (0.21)	0.80 (1.26)
eGFR (mL/min/1.73 m^2^)	*n*	171	19	116	28	5
	Baseline	69.8 (19.5)	100.3 (8.1)	73.1 (8.5)	51.2 (7.2)	19.2 (6.7)
	Week 52	67.8 (19.6)	93.0 (14.8)	71.5 (10.0)	50.7 (10.8)	17.4 (8.8)
	Change	−2.0 (7.2) ***	−7.2 (11.9) *	−1.5 (6.1) *	−0.5 (6.9)	−1.8 (4.9)
CK (U/L)	*n*	171	19	116	28	5
	Baseline	142.4 (142.3)	132.1 (61.5)	149.2 (168.0)	132.6 (60.5)	113.6 (34.4)
	Week 52	134.3 (88.4)	149.3 (113.0)	135.5 (89.6)	129.4 (75.8)	110.2 (29.5)
	Change	−8.1 (139.9)	17.2 (74.0)	−13.7 (163.9)	−3.2 (67.3)	−3.4 (15.1)

Data are presented as means (SD). Baseline eGFR categories are as follows: G1, eGFR ≥90 mL/min/1.73 m^2^; G2, eGFR ≥60 and <90 mL/min/1.73 m^2^; G3a–G3b, eGFR ≥30 and <60 mL/min/1.73 m^2^; G4–G5, eGFR <30 mL/min/1.73 m^2^. Three patients in hemodialysis were included in all participants but were excluded from the G4–G5 category. * *p* < 0.05, ** *p* < 0.01, *** *p* < 0.001 vs. baseline by Wilcoxon signed-rank test. eGFR, estimated glomerular filtration rate; AST, aspartate aminotransferase; ALT, alanine aminotransferase; γ-GT, γ-glutamyltransferase; ALP, alkaline phosphatase; CK, creatine kinase; SD, standard deviation.

## Abbreviations

ADR	Adverse drug reaction
AE	Adverse event
ALP	Akaline phosphatase
ALT	Alanine aminotransferase
Apo	Apolipoprotein
ASCVD	Atherosclerotic cardiovascular disease
AST	Aspartate aminotransferase
CK	Creatine kinase
CKD	Chronic kidney disease
CM	Chylomicron
CM-C	CM-cholesterol
CVD	Cardiovascular disease
eGFR	Estimated glomerular filtration rate
GCP	Good Clinical Practice
HbA1c	Hemoglobin A1c
HDL	High-density lipoprotein
HDL-C	HDL cholesterol
HPLC	High-performance liquid chromatography
hsCRP	High-sensitivity C-reactive protein
ICH	International Conference on Harmonisation
IL-1β	Interleukin-1β
JAPIC	Japan Pharmaceutical Information Center
KDIGO	Kidney Disease Improving Global Outcomes
LCAT	Lecithin-cholesterol acyltransferase
LC-MS-MS	Liquid chromatography-tandem mass spectrometry
LDL	Low-density lipoprotein
LDL-C	LDL cholesterol
LOCF	Last-observation-carried-forward
LPL	Lipoprotein lipase
PPARα	Peroxisome proliferator-activated receptor α
RemL-C	Remnant lipoprotein cholesterol
SAE	Serious adverse event
SD	Standard deviation
SPPARMα	Selective peroxisome proliferator-activated receptor-α modulator
TC	Total cholesterol
TG	Triglyceride
ULN	Upper limit of normal
VLDL	Very-low-density lipoprotein
VLDL-C	VLDL-cholesterol
γ-GT	γ-Glutamyltransferase
